# Long-Term Durability of High- and Very High-Power Short-Duration PVI by Invasive Remapping: The HPSD Remap Study

**DOI:** 10.1161/CIRCEP.123.012402

**Published:** 2024-01-29

**Authors:** Nándor Szegedi, Zoltán Salló, Vivien Klaudia Nagy, István Osztheimer, István Hizoh, Bálint Lakatos, Melinda Boussoussou, Gábor Orbán, Márton Boga, Arnold Béla Ferencz, Ferenc Komlósi, Patrik Tóth, Péter Perge, Attila Kovács, Béla Merkely, László Gellér

**Affiliations:** 1 Cardiology Department, Heart and Vascular Center, Semmelweis University, Budapest, Hungary.

**Keywords:** atrial fibrillation, catheter ablation, computed tomography angiography, fluoroscopy, pulmonary veins

## Abstract

**BACKGROUND:**

High-power short-duration ablation has shown impressive efficacy and safety for pulmonary vein isolation (PVI); however, initial efficacy results with very high power short-duration ablation were discouraging. This study compared the long-term durability of PVI performed with a 90- versus 50-W power setting.

**METHODS:**

Patients were randomized 1:1 to undergo PVI with the QDOT catheter using a power setting of 90 or 50 W. Three months after the index procedure, patients underwent a repeat electrophysiology study to identify pulmonary vein reconnections. Patients were followed for 12 months to detect AF recurrences.

**RESULTS:**

We included 46 patients (mean age, 64 years; women, 48%). Procedure (76 versus 84 minutes; *P* =0.02), left atrial dwell (63 versus 71 minutes; *P* =0.01), and radiofrequency (303 versus 1040 seconds; *P* <0.0001) times were shorter with 90- versus 50-W procedures, while the number of radiofrequency applications was higher with 90 versus 50 W (77 versus 67; *P* =0.01). There was no difference in first-pass isolation (83% versus 82%; *P* =1.0) or acute reconnection (4% versus 14%; *P* =0.3) rates between 90 and 50 W. Forty patients underwent a repeat electrophysiology study. Durable PVI on a per PV basis was present in 72/78 (92%) versus 68/77 (88%) PVs in the 90- and 50-W energy setting groups, respectively; effect size: 72/78−68/77=0.040, lower 95% CI=−0.051 (noninferiority limit=−0.1, ie, noninferiority is met). No complications occurred. There was no difference in 12-month atrial fibrillation-free survival between the 90- and 50-W groups (*P* =0.2).

**CONCLUSIONS:**

Similarly high rates of durable PVI and arrhythmia-free survival were achieved with 90 and 50 W. Procedure, left atrial dwell, and radiofrequency times were shorter with 90 W compared with 50 W. The sample size is too small to conclude the safety and long-term efficacy of the high and very high-power short-duration PVI; further studies are needed to address this topic.

**REGISTRATION:**

URL: https://www.clinicaltrials.gov; Unique identifier: NCT05459831.

**WHAT IS KNOWN?**

- Pulmonary vein isolation (PVI) is the cornerstone of atrial fibrillation ablation.
- High-power short-duration ablation has shown impressive efficacy and safety for PVI for atrial fibrillation ablation; however, initial efficacy results with very high-power short-duration ablation were discouraging.

**WHAT THE STUDY ADDS**

- In the high-power short-duration Remap study, acute procedural efficacy and 3-month durability of PVI were similarly high, indicating that a good quality PVI is achievable with both energy settings.
- On the other hand, procedural times are shorter with 90 versus 50 W, without affecting the acute and long-term procedural efficacy.
- Although the population in this study is one of the largest among remap studies published to date and no complications were observed with either the 90- or 50-W power setting, the sample size is too small to conclude the safety and long-term efficacy of the high and very high power short duration PVI; further studies are needed to confirm the safety of this technology in a larger population.

In the management of atrial fibrillation (AF), catheter ablation techniques have revolutionized treatment with established prognostic benefits. Pulmonary vein (PV) isolation (PVI) is the cornerstone of AF ablation,^1^ but despite current technological advancements, AF recurrence, primarily due to PV reconnection has remained an issue.^2–4^ PVI with high power and short duration (HPSD; >40 W) creates smaller but more uniform lesions compared to low-power long duration ablation, resulting in favorable safety and efficacy.^5–9^ Recently, a very HPSD (vHPSD; 90 W, 4 s) technique has been introduced, but initial efficacy results were mostly discouraging regarding first-pass isolation rates, acute reconnection rates, and arrhythmia-free survival.^10–14^

We hypothesized that vHPSD PVI could result in similar acute and long-term efficacy as ablation index-guided PVI with 50 W.^11^ Therefore, we designed the HPSD Remap study (Efficacy Comparison of High and Very High Power Short Duration Pulmonary Vein Isolation, URL: https://www.clinicaltrials.gov; unique identifier NCT05459831), in which durability of lesions was assessed by a repeat electrophysiology study after an index PVI performed with either a 90- or 50-W power setting.

## METHODS

### Study Design

The data that support the findings of this study are available from the corresponding author upon reasonable request. We conducted an investigator-initiated, single-center, prospective, randomized, noninferiority, parallel-group, open-label study to assess the long-term durability of PVI performed with 90- or 50-W power setting. Eligible patients had symptomatic paroxysmal or persistent AF (<12 months); full details of inclusion and exclusion criteria are listed in the study protocol (Supplemental Methods). All participants gave written informed consent. This study was conducted in accordance with Good Clinical Practice guidelines and the Declaration of Helsinki. Ethics approval was obtained from the Hungarian National Public Health and Medical Officer Service (9119-2/2022/EÜIG).

**Figure 1.**
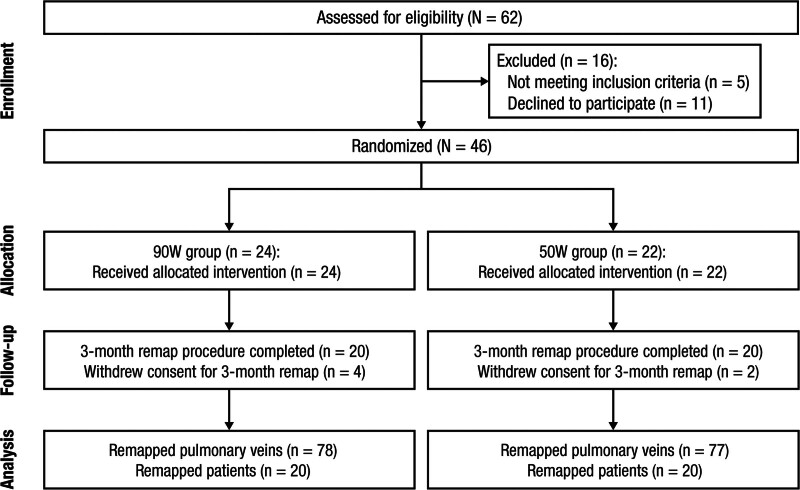
**Flowchart depicting study design, randomization, and follow-up.** Forty-six patients were randomized (1:1) to undergo pulmonary vein (PV) isolation (PVI) with very high power short-duration ablation (90 W, 4 s) or ablation index-guided PVI with a 50-W power setting. Three months after the index procedure, 40 patients underwent repeat left atrial (LA) high-density mapping to assess the long-term durability of ablation. The primary end point was PV reconnection at the 3-month remap procedure.

Forty-six patients were randomized in a 1:1 ratio to either undergo their first PVI procedure with vHPSD ablation (90 W, 4 s) or ablation index-guided PVI with a 50-W power setting. Randomization was performed using the www.studyrandomizer.com webpage. Three months after the index procedure, patients underwent repeat left atrial (LA) high-density mapping to assess the long-term durability of the ablation (Figure 1). The study period was between February 2022 and August 2023.

### Index Procedure

All patients underwent transthoracic echocardiography before the procedure. The left ventricular ejection fraction and LA volume index values were measured using the biplane Simpson method. Also, all patients underwent contrast-enhanced LA computed tomography angiography (CTA) by a 256-slice scanner (CardioGraphe, GE Healthcare) with prospective ECG-gated axial acquisition mode within 48 hours to exclude LA appendage thrombus. PV anatomy and diameter were analyzed on the cardiac CTA scans as previously described.^15^ If CTA was unable to exclude LA appendage thrombus, we performed transesophageal echocardiography for a definitive diagnosis.

**Figure 2.**
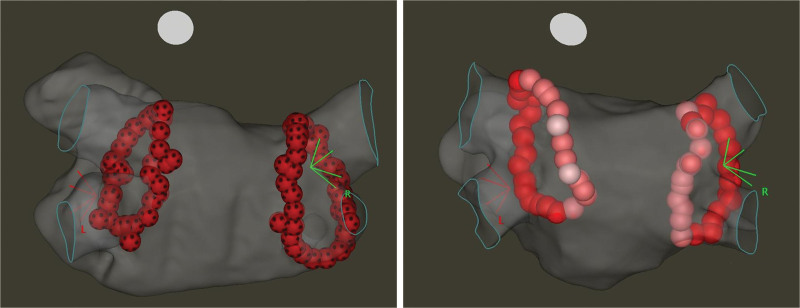
**CARTO3 electroanatomical map of pulmonary vein isolation (PVI) by randomization group (posteroanterior view). Left**, PVI performed with 90 W and 4 s setting, interlesion distance (ILD) <5 mm at each site. **Right**, PVI performed with 50 W energy and ablation index (AI; white tags indicate AI <400, pink tags AI 400–500, and red tags AI >500), ILD <5 mm at each site. L indicates left side; and R, right side.

Procedures were performed under conscious sedation using propofol and fentanyl. All patients were on non-vitamin K antagonist oral anticoagulants, a single dose of which was withheld on the morning of the procedure. Administration was resumed 4 hours after the ablation unless major bleeding events occurred. First, we obtained vascular access through the right femoral vein, then a double transseptal puncture was performed under fluoroscopy and pressure guidance. Intracardiac echocardiography was also used whenever it was necessary to perform a safe puncture. After the transseptal puncture, intravenous unfractionated heparin boluses were administered to maintain an activated clotting time of >300 s. A fast anatomic map of the LA was created with an electroanatomical mapping system (CARTO 3, Biosense Webster, Inc, Diamond Bar, CA), using a multipolar mapping catheter (either Lasso or PentaRay, Biosense Webster, Inc, Diamond Bar, CA). After that, a point-by-point radiofrequency PVI was performed with a steerable sheath (either Agilis, Abbott, Chicago, IL, or Vizigo, Biosense Webster, Inc, Diamond Bar, CA) and a contact-force sensing ablation catheter (QDOT Micro, Biosense Webster, Inc, Diamond Bar, CA) in temperature-controlled mode, according to the randomization group (Figure 2). No ablation beyond PVI was performed. Esophageal temperature monitoring was not used in this study.

For patients randomized to 90 W, all radiofrequency applications were delivered for 4 s (QMODE+). Interlesion distance was <5 mm at all sites, preferably <4 mm on the anterior wall. Target contact force was 5 to 40 g. The radius of the lesion tags was 3 mm.

For patients randomized to 50 W, ablation index-guided PVI was performed (QMODE) with the following protocol: interlesion distance <5 mm, target contact force 5 to 40 g, and target ablation index 500 on the anterior and 400 on the posterior LA wall. VisiTag settings were as follows: for location stability, the minimum time was 3 s, and the maximum range was 2.5 mm; force over time was 30%, with a minimum force of 3 g. The radius of the lesion tags was 3 mm.

During PV encirclement, the mapping catheter was positioned in the contralateral PVs to blind the operator to the presence or absence of PV potentials. After finishing the ablation circle according to the study protocol described above, first-pass isolation was assessed with the multipolar mapping catheter. If there was evidence of residual conduction into the PVs, touch-up applications were delivered with the same energy setting until complete isolation of all PVs was reached. Then, acute reconnection was assessed after a 20-minute waiting period for all PVs. In case of acute reconnection, further ablation was performed with the same energy setting, to isolate the veins.

The gap locations in the first-pass circle and segments of acute PV reconnection were registered on a 16-segment PV model.

### Repeat Electrophysiology Study

**Figure 3.**
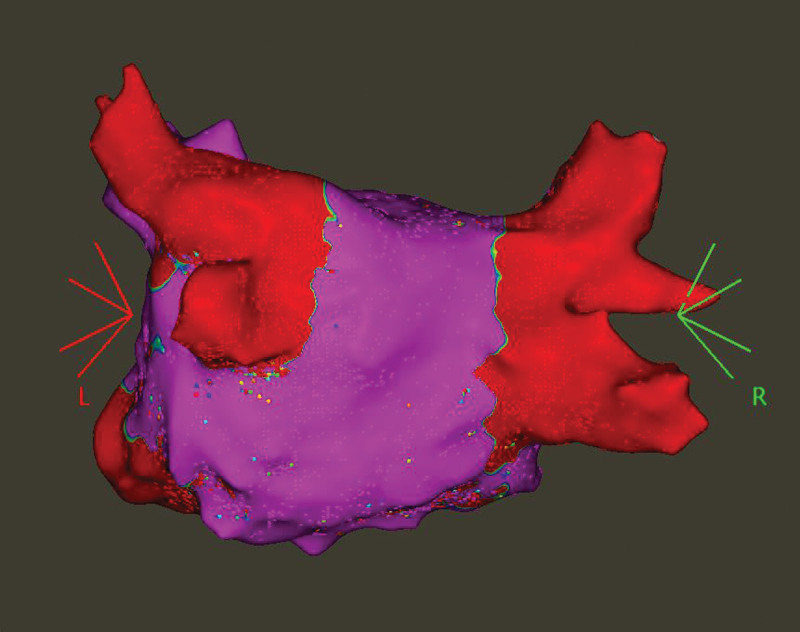
**High-density voltage map of a patient with 4 isolated pulmonary veins (PVs).** This voltage map was taken at the repeat procedure and shows the posteroanterior view taken with the CARTO3 mapping system. L indicates left side; and R, right side.

Patients underwent protocol-mandated repeat electrophysiology mapping 3 months after the index ablation, regardless of symptoms. Patients underwent transthoracic echocardiography and CTA before the repeat procedure. After femoral access and transseptal puncture, a high-density voltage and activation map during distal coronary sinus pacing were obtained with the CARTO3 mapping system using a multipolar catheter (PentaRay or OctaRay, Biosense Webster, Inc, Diamond Bar, CA). The LA-PVs were assessed for PV reconnection by this high-density map (minimum of 2000 points in an even distribution at all LA sites; Figure 3). The segments of PV reconnection were registered on a 16-segment PV model. In case of PV reconnection, the exact site(s) of PV-LA conduction were marked and then reablated with the QDOT Micro catheter with the same energy setting used for the index procedure.

### Follow-Up

A distinct 3-month follow-up was not necessary as patients were examined during the 3-month repeat procedure. All antiarrhythmic drugs were discontinued after the remap procedure. Clinical outpatient follow-ups were performed at 6 and 12 months after the index procedure, consisting of physical examination, 12-lead ECG, and 24-hour Holter ECG monitoring. Additional visits and ECG recordings were performed in case of arrhythmia symptoms. Adverse events occurring during the 12 months were also registered.

### End Points

The primary hypothesis was that PVI performed with 90 W would be noninferior to PVI with 50 W. The primary end point was PV reconnection at the 3-month repeat procedure. Secondary efficacy end points were as follows: first-pass isolation rate, acute reconnection rate, procedure time, LA dwell time, ablation time (the time from the first until the last radiofrequency application), radiofrequency time (time of total radiofrequency energy delivery), number of radiofrequency applications, amount of irrigation fluid, and fluoroscopy time and dose, and 12-month arrhythmia recurrence. The latter was defined as the recurrence of any documented atrial tachyarrhythmia >30 s after the 3-month blanking period. The secondary safety end point was the occurrence of any major procedure-related complication during the study period.

### Statistical Analysis

The study was designed using a noninferiority framework. Long-term durability results of PVI with 90 W power are lacking. Assuming a 7% rate of the primary end point (eg, reconnected PVs^9^) in both groups, 1:1 randomization, and a noninferiority margin of 10%, we calculated that 81 PVs per group would be required to have 80% power to test the noninferiority of 90- to 50-W energy setting, at a 1-sided alpha level of 0.05. Considering normal PV anatomy, this would require 40 remapped patients. Calculating with an anticipated 15% drop-out rate, we planned to enroll 46 patients. An interim analysis was planned after remapping 20-20 patients. The treatment arms were compared using the 1-sided 95% CI approach.

Normal or non-normal distribution of the parameters was tested with the Shapiro-Wilk test. Continuous variables are presented as mean and SD or median and interquartile range (25th–75th percentiles) where appropriate.

For secondary end points, superiority analysis was performed. Student *t* test or the Mann-Whitney *U* test was used for unpaired group comparison. Categorical variables are presented as frequency and percentage and were compared by Fisher’s exact test. Statistical analyses were performed using IBM SPSS 25 (Apache Software Foundation) and GraphPad Prism 8 (GraphPad Softwares, Inc) software products, and with R version 4.3.1 (R Foundation for Statistical Computing, Vienna, Austria) using the rms version 6.7-1 additional packages. A 2-tailed *P* value of <0.05 was considered statistically significant. No subgroup analysis was planned.

## RESULTS

### Baseline Characteristics

Overall, 46 patients were enrolled in the study, all of them underwent the index ablation procedure according to the randomization. Baseline characteristics are shown in Table 1. Mean (SD) age was 64 (8) years, 22 were women, and 26 had paroxysmal AF. The most frequent comorbidities were hypertension (n=35) and diabetes (n=15).

**Table 1.**
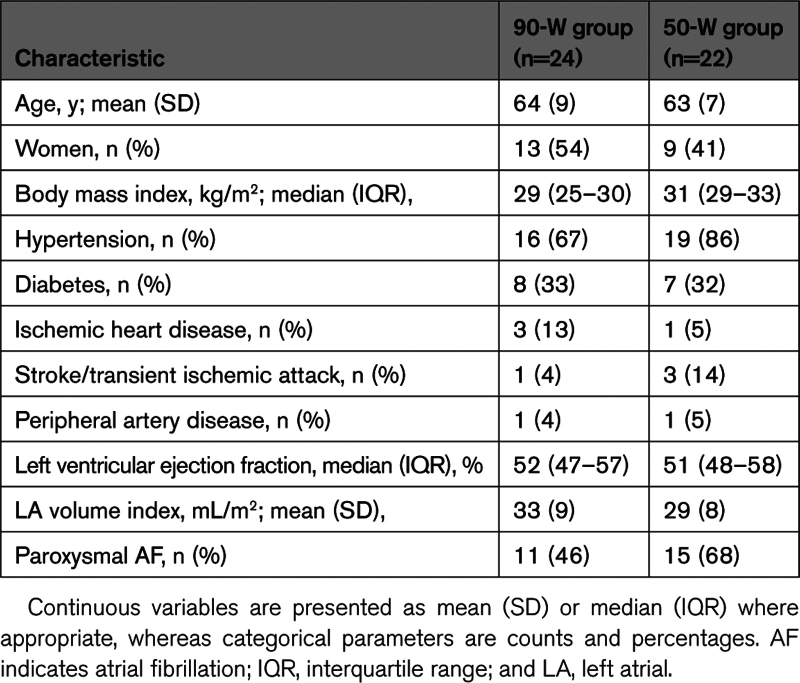
Baseline Characteristics of the Study Population

### Index Procedure

**Figure 4.**
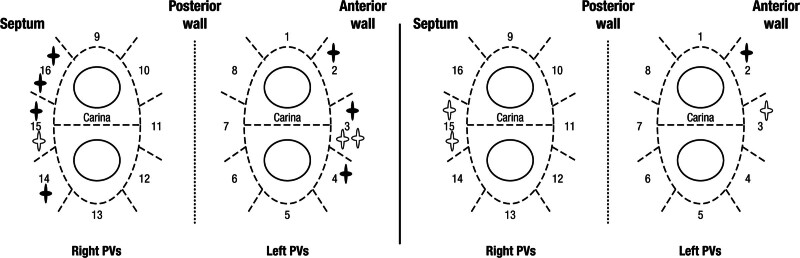
**Distribution of gaps in first-pass encirclement and acute reconnection sites.** Distribution of gaps in first-pass encirclement are shown in the left panel, and sites of acute reconnection are shown in the right panel on the 16-segment pulmonary veins (PV) model. Bold stars indicate gaps of the 90-W group, while empty stars indicate gaps of the 50-W group.

The parameters of the index procedure are shown in Table 2. All PVs were successfully isolated at the end of all index procedures. There was no difference in first-pass isolation rate (20 versus 18; *P*=1.0) and acute reconnection rate (1 versus 3; *P*=0.3) between patients ablated with 90 W compared with 50 W. Procedure time (76 versus 84 minutes; *P*=0.02), LA dwell time (63 versus 71 minutes; *P*=0.01), ablation time (27 versus 34 minutes; *P*=0.01), and radiofrequency time (303 versus 1040 s; *P*<0.0001) were shorter and the amount of irrigation fluid was lower (189 versus 248 mL; *P*=0.01) in the 90-W group compared with the 50-W group. The distribution of gaps in the first-pass circle and locations of acute reconnection are shown in Figure 4.

**Table 2.**
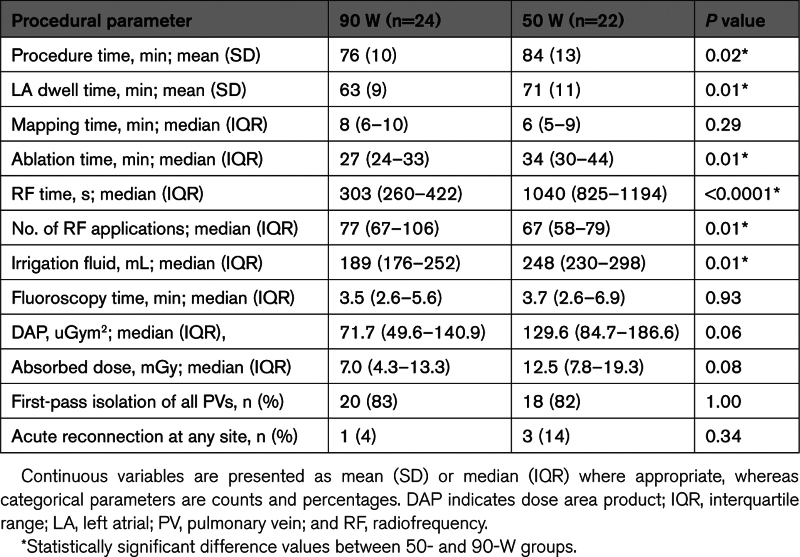
Parameters of the Index Procedure

### Repeat Electrophysiology Study

Six patients withdrew their consent from the repeat procedure. Forty patients (20 in each treatment arm) underwent the repeat electrophysiology study after a median of 91 (interquartile range, 91–98) days. Before the procedure, LA CTA was repeated to evaluate the potential PV narrowing. No significant PV stenosis occurred.

**Figure 5.**
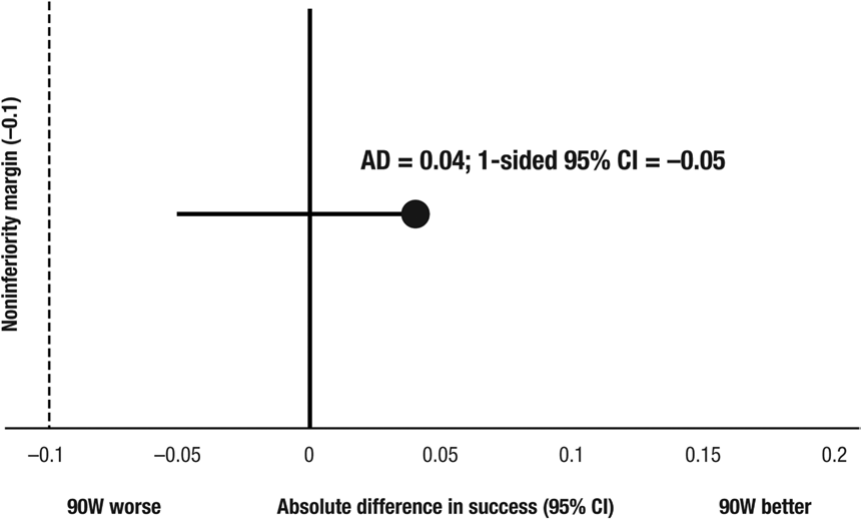
**Primary end point analysis.** Pulmonary vein isolation with 90 W is noninferior to 50 W with respect to pulmonary vein reconnections at the 3-month remap procedure.

Durable pulmonary vein isolation on a per PV basis was present in 72/78 (92%) versus 68/77 (88%) PVs in the 90- and 50-W energy setting groups, respectively. Effect size: 72/78 to 68/77=0.040, lower 95% CI=−0.051 (noninferiority limit=−0.1, eg, noninferiority is met; Figure 5). An interim analysis after the completion of the remap of these 20-20 patients in each group showed that the observed durable pulmonary vein isolation rate was lower in the 50W group (88%) and higher in the 90-W group (92%), and that with these rates the noninferiority was met (a posteriori power, 0.904).

**Figure 6.**
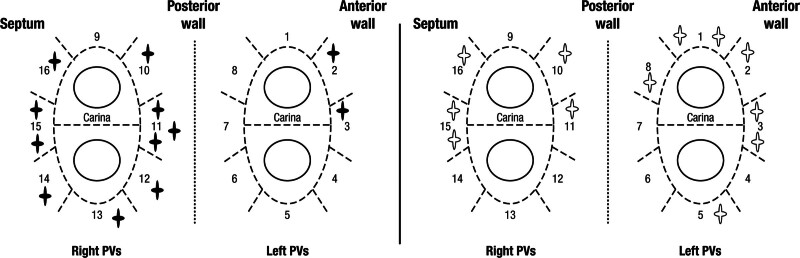
**Distribution of late pulmonary veins (PV) reconnection sites with 90 and 50 W.** Distribution of the late PV reconnection sites with 90 W are shown in the left panel as bold stars and with 50 W are shown in the right panel as empty stars on the 16-segment PV model.

Durable isolation of all PVs on a per-patient basis was present in 29 of 40 patients (72.5%), without any significant difference between the 90- and 50-W groups (16 [80%] versus 13 [65%], respectively; *P*=0.48). On a per-segment basis, there was also no difference between the 90- and 50-W groups (12/320 versus 12/320 reconnected segments, respectively; *P*=1.0). The distribution of late PV reconnection sites is shown in Figure 6. All reconnected PVs were successfully reisolated.

No major complications occurred in any of the 86 procedures. No audible steam pops occurred.

### 12-Month Follow-Up

No patient was lost to follow-up. Atrial tachyarrhythmia recurrence was documented in 6/46 (13%) patients; 3 of these patients had a paroxysmal arrhythmia pattern and 3 had a persistent arrhythmia pattern (Table S1). Of the patients with atrial tachyarrhythmia recurrence, 5 were ablated with 90 W and 1 with 50 W; there was no significant difference between the 2 groups (*P*=0.2). Four of these patients had all PVs isolated during the remap procedure. The median (interquartile range) time to AT recurrence was 9.5 (5.75–11.25) months.

## DISCUSSION

### Main Findings

PVI with both 90- and 50-W ablation results in impressive acute procedural efficacy, long-term durability, and a low rate of arrhythmia recurrence. PVI with vHPSD ablation (90 W, 4 s) is associated with shorter procedure, LA dwell, ablation, and radiofrequency times compared with the 50-W power setting.

### vHPSD PVI

The durability of electrical PVI is the most meaningful procedural end point in AF ablation procedures. After ablation, the primary mechanism of recurrence is electrical PV reconnection.^4^ Previous studies of high power ablation (40–50 W) showed good efficacy and a favorable safety profile, most likely because of the higher likelihood of a stable catheter position during the shorter radiofrequency applications.^16,17^

The vHPSD ablation technique (90 W, 4 s) was recently introduced. The QDOT Micro catheter enables a unique temperature control mode of radiofrequency energy delivery with accurate surface temperature measurement. Preclinical data were encouraging as vHPSD resulted in improved linear continuity, shorter ablation time, and a good safety profile. Although vHPSD ablation results in a different lesion geometry and smaller size compared with conventional lower-power ablation, the depth of the vHPSD applications is mostly sufficient to create transmural lesions in the left atrium.^5–8^ These properties of vHPSD lesions raise a need for decreasing the interlesion distance, verified in a clinical setting by our previous report.^11^

The advantages of this novel technique have also translated into clinically meaningful results in previous studies, as evidenced by substantially shorter total procedure, LA ablation, and radiofrequency times compared with ablation with lower power.^11,13^ Our current study strongly underpins these findings, as procedures performed with 90 W were shorter than those with 50 W. Nevertheless, acute procedural efficacy outcome measures of vHPSD PVI have previously shown a huge variety; for example, first-pass isolation rates ranged from 18% to 80% and acute reconnection rates ranged from 27% to 32%.^10,11,13^ Studies that showed low first-pass isolation and high acute reconnection rates used larger (<6 mm) inter-tag distance, which explains the difference from our current results.^10,14,18^ Recently published papers show similar safety and 1-year follow-up results with 90- and 25 to 50-W energy settings.^11,14,18–20^ Although AF recurrence is a gold-standard measure of the efficacy of PVI, it is still not the most objective end point of procedural success, as there is not a one-to-one relationship between PV reconnection and AF recurrence. Invasive repeat LA mapping is considered the most accurate way to evaluate the durability of PVI and, thus, to validate a given technique’s efficacy for PVI.

### Comparison With Previous Remap Studies

We acknowledge that trials that mandate invasive remapping procedures regardless of arrhythmia recurrence are challenging to conduct as asymptomatic patients’ willingness to undergo a second procedure might be low. Thus, most data on chronic PVI durability result from studies where repeat procedure was only performed in case of recurrent symptoms, which does not provide accurate feedback on the overall durability of PVI.^4,21–23^

A few studies are available in which an intentional, protocol-mandated invasive repeat procedure was performed to assess the durability of the PVI. The published rates of durable isolation on a per-vein and per-patient basis range widely. The main results of the remap studies is summarized in Table 3.^9,24–28^

**Table 3.**
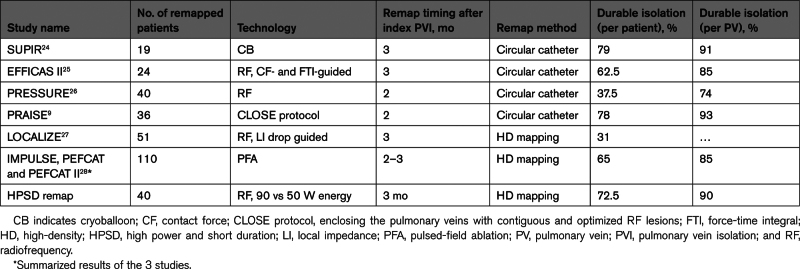
Summary of Studies Evaluating PVI’s Efficacy With Invasive Left Atrial Remap

In the current study, 40 patients underwent initial PVI either with 50 or with 90 W for 4 s and completed the 3-month remap procedure. Despite the complexity of the study design arising from the mandatory repeat procedure, we included a substantial number of patients, most of whom underwent the second procedure. At 3 months, the PVs of these 40 patients were assessed with a high-density left atrial remap, showing durable PVI of all PVs in 72.5% of patients (80% with 90 W and 65% with 50 W) and 90% of PVs (92% with 90 W and 88% with 50 W). Our results are similar to the PRAISE study results, where the index procedure was performed with a similarly advanced ablation tool and protocol.^9^ Of note, we performed the repeat procedure at 3 months compared with 2 months in the PRAISE study. Moreover, we used high-density mapping for the repeat procedure after initial radiofrequency ablation, which might enhance the detection of antral reconnection sites. Additionally, the location and distribution of acute gaps and reconnections as well as chronic gaps observed in the current study differ from those observed in prior studies, as we have found the right carina to the most frequent site of reconnection with radiofrequency ablation.^29,30^ No complications occurred during our study.

### Clinical Implications

Our results indicate that in contrast to previous studies with low first-pass isolation rates, favorable efficacy is achievable with vHPSD PVI (90 W, 4 s). The first-pass isolation and acute and chronic reconnection rates were similar to our comparator group, ablated with 50 W and ablation index guidance, and similar to previously published data. This result could be achieved with a meticulous PVI protocol, where all 90-W applications lasted 4 s, and the interlesion distance was <5 mm at all sites (preferably <4 mm anteriorly). Important to note, as VisiTag and ablation index are not available for 90-W applications, there is no accurate feedback on lesion quality. On the contrary, ablation points seem at the location where the application was started, regardless of the contact force and stability. This requires attention from the operating physician to maintain sufficient catheter-tissue contact and consider an additional overlapping application if poor stability or contact force is observed during the energy delivery.

### Study Limitations

We acknowledge that our study has certain limitations. First, it was a single-center study with 4 operating physicians (each with experience of >300 PVIs). We included both paroxysmal and persistent AF patients; the left atrial size of the studied patients was normal. No complications occurred in our study population during the 86 procedures; however, given the relatively low sample size and low incidence of complications, this study was underpowered to evaluate the safety profile of HPSD and vHPSD PVI. Further studies are needed to address the safety of this technology.

### Conclusions

We demonstrate that, besides impressive acute procedural efficacy, the long-term durability of PVI and 12-month AF-free survival was similarly high, both with 90- and 50-W power settings. Procedure, LA dwell, and radiofrequency times were shorter with a 90-W power setting compared with 50 W. The sample size is too small to conclude the safety and long-term efficacy of the HPSD and vHPSD PVI; further studies are needed to confirm the safety of this technology in a larger population.

## ARTICLE INFORMATION

### Acknowledgments

The authors thank Brigitta Rusznák, Károly Ladunga, and Gergely Nagy for their contributions to the study conduct. Editorial assistance was provided in accordance with Good Publication Practice (GPP 2022) guidelines by Michelle Hughes, PhD of Lumanity Communications, Inc (Yardley, PA) and was funded by Biosense Webster Inc. (Irvine, CA).

### Sources of Funding

This research was funded as grant ID IIS-653 through Biosense Webster’s Investigator-Initiated Study program. This study was supported by the National Research, Development and Innovation Office of Hungary (NKFIA; NVKP_16-1-2016-0017 National Heart Program). Project no. RRF-2.3.1-21-2022-00003 has been implemented with the support provided by the European Union. This research work was conducted with the support of the National Academy of Scientist Education Program of the National Biomedical Foundation under the sponsorship of the Hungarian Ministry of Culture and Innovation (FEIF/646-4/2021-ITM_SZERZ). Melinda Boussoussou was supported by the ÚNKP-22-3-II-SE (ÚNKP-22-3-II-SE-51), New National Excellence Program of the Ministry for Innovation and Technology from the source of the National research, Development and Innovation fund. Drs Szegedi and Orbán were supported by the MD-PhD Excellence Program of Semmelweis University (EFOP-3.6.3-VEKOP-16-2017-00009) and by the ÚNKP-22-2-III-SE-59 New National Excellence Program of the Ministry for Culture and Innovation from the source of the National Research, Development and Innovation Fund. Editorial assistance for this article was funded by Biosense Webster, Inc. (Irvine, CA).

### Disclosures

Drs Szegedi and Gellér report consulting fees from Biosense Webster, Abbott, and Boston Scientific, not related to the present study. Drs Salló and Nagy report consulting fees from Abbott and Boston Scientific, not related to the present study. Drs Lakatos and Kovács report personal fees from Argus Cognitive, Inc, outside the submitted work. The other authors report no conflicts.

### Supplemental Material

Supplemental Methods

Supplemental Table S1

## Supplementary Material

## References

[1] Hindricks G Potpara T Dagres N Arbelo E Bax JJ Blomstrom-Lundqvist C Boriani G Castella M Dan GA Dilaveris PE; ESC Scientific Document Group. 2020 ESC Guidelines for the diagnosis and management of atrial fibrillation developed in collaboration with the European Association for Cardio-Thoracic Surgery (EACTS): The Task Force for the diagnosis and management of atrial fibrillation of the European Society of Cardiology (ESC) Developed with the special contribution of the European Heart Rhythm Association (EHRA) of the ESC. Eur Heart J. 2021;42:373–498. doi: 10.1093/eurheartj/ehaa612 32860505 10.1093/eurheartj/ehaa612

[2] Taghji P El Haddad M Phlips T Wolf M Knecht S Vandekerckhove Y Tavernier R Nakagawa H Duytschaever M. Evaluation of a strategy aiming to enclose the pulmonary veins with contiguous and optimized radiofrequency lesions in paroxysmal atrial fibrillation: a pilot study. JACC Clin Electrophysiol. 2018;4:99–108. doi: 10.1016/j.jacep.2017.06.023 29600792 10.1016/j.jacep.2017.06.023

[3] Szegedi N Salló Z Perge P Piros K Nagy VK Osztheimer I Merkely B Geller L. The role of local impedance drop in the acute lesion efficacy during pulmonary vein isolation performed with a new contact force sensing catheter—a pilot study. PLoS One. 2021;16:e0257050. doi: 10.1371/journal.pone.0257050 34529678 10.1371/journal.pone.0257050 PMC8445471

[4] De Pooter J Strisciuglio T El Haddad M Wolf M Phlips T Vandekerckhove Y Tavernier R Knecht S Duytschaever M. Pulmonary vein reconnection no longer occurs in the majority of patients after a single pulmonary vein isolation procedure. JACC Clin Electrophysiol. 2019;5:295–305. doi: 10.1016/j.jacep.2018.11.020 30898231 10.1016/j.jacep.2018.11.020

[5] Nakagawa H Ikeda A Sharma T Govari A Ashton J Maffre J Lifshitz A Fuimaono K Yokoyama K Wittkampf FHM. Comparison of in vivo tissue temperature profile and lesion geometry for radiofrequency ablation with high power-short duration and moderate power–moderate duration: effects of thermal latency and contact force on lesion formation. Circ Arrhythm Electrophysiol. 2021;14:e009899. doi: 10.1161/CIRCEP.121.009899 34138641 10.1161/CIRCEP.121.009899

[6] Leshem E Zilberman I Tschabrunn CM Barkagan M Contreras-Valdes FM Govari A Anter E. High-power and short-duration ablation for pulmonary vein isolation: biophysical characterization. JACC Clin Electrophysiol. 2018;4:467–479. doi: 10.1016/j.jacep.2017.11.018 30067486 10.1016/j.jacep.2017.11.018

[7] Barkagan M Contreras-Valdes FM Leshem E Buxton AE Nakagawa H Anter E. High-power and short-duration ablation for pulmonary vein isolation: safety, efficacy, and long-term durability. J Cardiovasc Electrophysiol. 2018;29:1287–1296. doi: 10.1111/jce.13651 29846987 10.1111/jce.13651

[8] Lozano-Granero C Franco E Matía-Francés R Hernandez-Madrid A Sanchez-Perez I Zamorano JL Moreno J. Characterization of high-power and very-high-power short-duration radiofrequency lesions performed with a new-generation catheter and a temperature-control ablation mode. J Cardiovasc Electrophysiol. 2022;33:2528–2537. doi: 10.1111/jce.15676 36116038 10.1111/jce.15676

[9] Hussein A Das M Riva S Morgan M Ronayne C Sahni A Shaw M Todd D Hall M Modi S. Use of ablation index-guided ablation results in high rates of durable pulmonary vein isolation and freedom from arrhythmia in persistent atrial fibrillation patients: the PRAISE study results. Circ Arrhythm Electrophysiol. 2018;11:e006576. doi: 10.1161/CIRCEP.118.006576 30354288 10.1161/CIRCEP.118.006576

[10] Mueller J Halbfass P Sonne K Nentwich K Ene E Berkovitz A Lehmkuhl L Barth S Simu GR Waechter C. Safety aspects of very high power very short duration atrial fibrillation ablation using a modified radiofrequency RF-generator: single-center experience. J Cardiovasc Electrophysiol. 2022;33:920–927. doi: 10.1111/jce.15433 35233883 10.1111/jce.15433

[11] Salló Z Perge P Balogi B Orban G Piros K Herczeg S Nagy KV Osztheimer I Abraham P Merkely B. Impact of high-power and very high-power short-duration radiofrequency ablation on procedure characteristics and first-pass isolation during pulmonary vein isolation. Front Cardiovasc Med. 2022;9:935705. doi: 10.3389/fcvm.2022.935705 35872909 10.3389/fcvm.2022.935705 PMC9300971

[12] Orbán G Salló Z Perge P Abraham P Piros K Nagy KV Osztheimer I Merkely B Geller L Szegedi N. Characteristics of very high-power, short-duration radiofrequency applications. Front Cardiovasc Med. 2022;9:941434. doi: 10.3389/fcvm.2022.941434 35911564 10.3389/fcvm.2022.941434 PMC9326019

[13] Reddy VY Grimaldi M De Potter T Vijgen JM Bulava A Duytschaever MF Martinek M Natale A Knecht S Neuzil P. Pulmonary vein isolation with very high power–short duration temperature-controlled lesions: the QDOT-FAST trial. JACC Clin Electrophysiol. 2019;5:778–786. doi: 10.1016/j.jacep.2019.04.009 31320006 10.1016/j.jacep.2019.04.009

[14] Mueller J Nentwich K Ene E Berkovitz A Sonne K Chakarov I Barth S Waechter C Behnes M Akin I. Radiofrequency ablation of atrial fibrillation—50 W or 90 W? J Cardiovasc Electrophysiol. 2022;33:2504–2513. doi: 10.1111/jce.15681 36124396 10.1111/jce.15681

[15] Szegedi N Vecsey-Nagy M Simon J Szilveszter B Herczeg S Kolossvary M Idelbi H Osztheimer I Klaudia Nagy V Tahin T. Orientation of the right superior pulmonary vein affects outcome after pulmonary vein isolation. Eur Heart J Cardiovasc Imaging. 2022;23:515–523. doi: 10.1093/ehjci/jeab041 33693618 10.1093/ehjci/jeab041

[16] Kewcharoen J Techorueangwiwat C Kanitsoraphan C Leesutipornchai T Akoum N Bunch TJ Navaravong L. High-power short duration and low-power long duration in atrial fibrillation ablation: a meta-analysis. J Cardiovasc Electrophysiol. 2021;32:71–82. doi: 10.1111/jce.14806 33155303 10.1111/jce.14806

[17] Kaneshiro T Kamioka M Hijioka N Yamada S Yokokawa T Misaka T Hikichi T Yoshihisa A Takeishi Y. Characteristics of esophageal injury in ablation of atrial fibrillation using a high-power short-duration setting. Circ Arrhythm Electrophysiol. 2020;13:e008602. doi: 10.1161/CIRCEP.120.008602 32915644 10.1161/CIRCEP.120.008602

[18] Bortone A Albenque JP Ramirez FD Haissaguerre M Combes S Constantin M Laborie G Brault-Noble G Marijon E Jais P. 90 vs 50-watt radiofrequency applications for pulmonary vein isolation: experimental and clinical findings. Circ Arrhythm Electrophysiol. 2022;15:e010663. doi: 10.1161/CIRCEP.121.010663 35363039 10.1161/CIRCEP.121.010663

[19] Osorio J Hussein AA Delaughter MC Monir G Natale A Dukkipati S Oza S Daoud E Di Biase L Mansour M; Q-FFICIENCY Trial Investigators. Very high-power short-duration, temperature-controlled radiofrequency ablation in paroxysmal atrial fibrillation: the prospective multicenter Q-FFICIENCY trial. JACC Clin Electrophysiol. 2023;9:468–480. doi: 10.1016/j.jacep.2022.10.019 36752484 10.1016/j.jacep.2022.10.019

[20] O’Neill L El Haddad M Berte B Kobza R Hilfiker G Scherr D Manninger M Wijnmaalen AP Trines SA Wielandts JY. Very high-power ablation for contiguous pulmonary vein isolation: results from the randomized POWER PLUS trial. JACC Clin Electrophysiol. 2023;9:511–522. doi: 10.1016/j.jacep.2022.10.039 36752467 10.1016/j.jacep.2022.10.039

[21] Miyazaki S Taniguchi H Hachiya H Nakamura H Takagi T Hirao K Iesaka Y. Clinical recurrence and electrical pulmonary vein reconnections after second-generation cryoballoon ablation. Heart Rhythm. 2016;13:1852–1857. doi: 10.1016/j.hrthm.2016.05.025 27241352 10.1016/j.hrthm.2016.05.025

[22] Chen S Schmidt B Bordignon S Perrotta L Bologna F Chun KRJ. Impact of cryoballoon freeze duration on long-term durability of pulmonary vein isolation: ICE Re-Map study. JACC Clin Electrophysiol. 2019;5:551–559. doi: 10.1016/j.jacep.2019.03.012 31122376 10.1016/j.jacep.2019.03.012

[23] Francke A Scharfe F Schoen S Wunderlich C Christoph M. Reconnection patterns after CLOSE-guided 50 W high-power-short-duration circumferential pulmonary vein isolation and substrate modification—PV reconnection might no longer be an issue. J Cardiovasc Electrophysiol. 2022;33:1136–1145. doi: 10.1111/jce.15396 35118734 10.1111/jce.15396

[24] Reddy VY Sediva L Petru J Skoda J Chovanec M Chitovova Z Di Stefano P Rubin E Dukkipati S Neuzil P. Durability of pulmonary vein isolation with cryoballoon ablation: results from the Sustained PV Isolation with Arctic Front Advance (SUPIR) study. J Cardiovasc Electrophysiol. 2015;26:493–500. doi: 10.1111/jce.12626 25644659 10.1111/jce.12626

[25] Kautzner J Neuzil P Lambert H Peichl P Petru J Cihak R Skoda J Wichterle D Wissner E Yulzari A. EFFICAS II: optimization of catheter contact force improves outcome of pulmonary vein isolation for paroxysmal atrial fibrillation. Europace. 2015;17:1229–1235. doi: 10.1093/europace/euv057 26041872 10.1093/europace/euv057 PMC4535556

[26] Das M Wynn GJ Saeed Y Gomes S Morgan M Ronayne C Bonnett LJ Waktare JEP Todd DM Hall MCS. Pulmonary vein re-isolation as a routine strategy regardless of symptoms: the PRESSURE randomized controlled trial. JACC Clin Electrophysiol. 2017;3:602–611. doi: 10.1016/j.jacep.2017.01.016 29759434 10.1016/j.jacep.2017.01.016

[27] García-Bolao I Ramos P Luik A Sulkin MS Gutbrod SR Oesterlein T Laughner JI Richards E Meyer C Yue A. Local impedance drop predicts durable conduction block in patients with paroxysmal atrial fibrillation. JACC Clin Electrophysiol. 2022;8:595–604. doi: 10.1016/j.jacep.2022.01.009 35589172 10.1016/j.jacep.2022.01.009

[28] Reddy VY Dukkipati SR Neuzil P Anic A Petru J Funasako M Cochet H Minami K Breskovic T Sikiric I. Pulsed field ablation of paroxysmal atrial fibrillation: 1-year outcomes of IMPULSE, PEFCAT, and PEFCAT II. JACC Clin Electrophysiol. 2021;7:614–627. doi: 10.1016/j.jacep.2021.02.014 33933412 10.1016/j.jacep.2021.02.014

[29] Chinitz JS Kapur S Barbhaiya C Kumar S John R Epstein LM Tedrow U Stevenson WG Michaud GF. Sites with small impedance decrease during catheter ablation for atrial fibrillation are associated with recovery of pulmonary vein conduction. J Cardiovasc Electrophysiol. 2016;27:1390–1398. doi: 10.1111/jce.13095 27581553 10.1111/jce.13095

[30] Sorensen SK Johannessen A Worck R Hansen ML Ruwald MH Hansen J. Differential gap location after radiofrequency versus cryoballoon pulmonary vein isolation: Insights from a randomized trial with protocol-mandated repeat procedure. J Cardiovasc Electrophysiol. 2023;34:519–526. doi: 10.1111/jce.15821 36640430 10.1111/jce.15821

