# Supplementary material for: Long-Term Durability of High- and Very High-Power Short-Duration PVI by Invasive Remapping: The HPSD Remap Study
Source: Circ Arrhythm Electrophysiol. 2024 Jan 29;17(2):e012402. doi: 10.1161/CIRCEP.123.012402 (PMC10876176; doi:10.1161/CIRCEP.123.012402)
Supplement: Supplementary file 1 [file hae-17-e012402-s001.pdf]

SUPPLEMENTAL MATERIAL

Table of Contents

**Supplemental Material**	**Page**
Supplemental Methods: Study protocol: Efficacy comparison of high and very high power short duration pulmonary vein isolation	2
Supplemental Table 1: 12-Month AF Recurrences	7

Supplemental Methods

Study Protocol

Efficacy comparison of high and very high power short duration pulmonary vein isolation

1. **Background**

   Atrial fibrillation (AF) is the most common sustained cardiac arrhythmia in adults. Its prevalence increases with age, affecting 3/100 people, and its incidence is also correlated with age, affecting 30/100,000 people per year. Its clinical significance stems from common complications (stroke, heart failure) and concomitant higher mortality.

   The most effective treatment method of atrial fibrillation is catheter ablation, which seeks to maintain sinus rhythm. Isolation of the triggering pulmonary veins (PVI) from the left atrium is the cornerstone of AF ablation. PVI has undergone significant technical development over the past decade; however, the chances of arrhythmia recurrence and the occurrence of procedural complications are not negligible.

   Experiments have shown that lesions with a larger surface diameter but less depth develop during HPSD ablation; the higher the energy is, the shallower and wider the lesions are. These lesion properties make it easier to create a solid line from the ablation points, while the shallower lesion causes less damage to the surrounding tissues. The latest application of HPSD technology is the so-called “very high power short duration” (vHPSD) ablation when we apply 90W power. No data are available on long-term efficacy or safety comparing ablation with HPSD vs. vHPSD.

2. **Objective**: To evaluate the durability of radiofrequency PVI performed with 90W (QMODE plus) and 50W (QMODE) with the QDot catheter.

3. **Primary hypothesis**: Pulmonary vein isolation is equally highly effective when performed with 50W guided by ablation index (QMODE) and inter-tag distance <5 mm and when performed with 90W (QMODE+) and inter-tag distance <5 mm.
4. **Study Design**: Single-center, prospective, randomized study. Patients will be randomly assigned in a 1:1 ratio to HPSD (50W) and vHPSD (90W) PVI.
5. **Research period**: Planned research period from the second quarter of 2022 for a maximum of 1 year.
6. **Study subjects**

- Planned number of included subjects: 40.
- *Inclusion criteria*
    - Symptomatic paroxysmal/persistent AF
    - Age >18 years
    - Willingness to sign informed consent form
- *Exclusion criteria*
    - Contraindication to ablation
    - Contraindication of long-term anticoagulation
    - Long-standing persistent AF
    - History of PVI
    - History of cardiac surgery
    - Pregnancy
    - Active malignancy
    - Life expectancy <1 year
    - Valvular AF
    - Reversible cause of AF (e.g. hyperthyroidism).

7. **Study procedures**:

- Initial PVI will be performed via femoral access after double transseptal puncture guided by fluoroscopy and pressure monitoring. If necessary ICE will be used for the transseptal puncture. A fast anatomical left atrial map will be created with a multipolar mapping catheter; then, point-by-point PVI will be performed with QDot catheter with 50W or 90W according to randomization. Procedures with 50W will be guided by ablation index (400 posteriorly and 500 anteriorly) and an inter-tag distance of <5 mm will be used. For 90W procedures, an inter-tag distance of <5 mm will be used everywhere but preferably <4 mm is desirable on the anterior wall. After creating the isolation circle, the presence or absence of first-pass isolation will be assessed by multipolar catheters. If PVI is not complete at this point, it will be completed to reach complete isolation of all veins. After that, a 20 minutes waiting period will be the next step to evaluate presence or absence of acute reconnection of the PV-s. In case of acute reconnection, touch-up applications will be delivered to complete the PVI.
- Three months after the initial procedure, patients will undergo a repeated high density left atrial mapping with PentaRay or, if available OctaRay catheter to evaluate the durability of the PVI. Exact gap numbers and locations will be registered (based on the 16 segment PV model). If necessary, rePVI will be performed (it is recommended to use the same energy setting as for the index procedure).

8. **Endpoints**:

- Primary endpoint: reconnection of pulmonary vein(s) to the left atrium at 3 month
- Secondary endpoints:
    - Procedure time of the initial PVI
    - Fluoroscopy time and dose of the initial PVI
    - Major complication of the initial PVI
    - First pass isolation during the initial PVI

- Acute reconnection during the initial PVI
- Major complication of the second (remapping) procedure
- 12-month AF recurrence.

The followings are considered major complications:

- Complications involving the vascular access site: femoral pseudoaneurysm / arterio-venous fistula / hematoma with bleeding requiring blood transfusion
- Pericardial tamponade
- Stroke / TIA
- Esophageal injury / perforation / fistula
- Myocardial infarction
- Phrenic nerve paresis
- Pulmonary vein stenosis
- Conduction system damage requiring pacemaker implantation
- Death.

9. Statistical analysis

Continuous variables are expressed as mean and standard deviation, or median and interquartile ranges, depending on the normal of non-normal distribution. Categorical variables are expressed in numbers and percentages. Continuous variables are compared by parametric or non-parametric tests, depending on the distribution, while categorical variables are compared by Chi-square test or Fisher's exact test. The predictive value of different ablation techniques in outcome is planned to be analyzed by logistic regression. A two-tailed p-value of <0.05 will be considered statistically significant. Statistical analyses will be performed using IBM SPSS 25 (Apache Software Foundation, USA) and GraphPad Prism 7.1 (GraphPad Softwares Inc., USA) software products.

10. Data management

All study patient data is entered into Semmelweis University's electronic database (accessible only from Semmelweis University's network) in a coded and unique manner, with a unique identifier, to which project staff has a password-protected, defined level of access. Each person involved in the study is given a unique identification code, and the data stored in the database is linked to that unique identifier. Thus, a data set will be incomprehensible and unusable for an external (unauthorized) user. The data belonging to the unique identification code, with which the patient's identity can be clearly indicated (name, place and date of birth, clinical reference number, identification number, identity card number, etc.) are not available from the database and are stored separately from it. Access to personal data is restricted to institutional leaders and program professional leaders.

11. Personal and material requirements

The personal and material requirements of the research are available at the Heart and Vascular Center of Semmelweis University. The personal requirements are given by the staff of the Electrophysiological Laboratory, and the material conditions are provided by the equipment of the same laboratory.

12. Funding: A research grant application is submitted to the Johnson and Johnson Company.

13. Ethics approval: An ethics approval was obtained from the Hungarian National Public Health Center (No. 8119-2/2022/EÜIG).

Supplemental Table I. 12-Month AF Recurrences

Arrhythmia recurrence was defined as the recurrence of any documented atrial tachyarrhythmia >30 seconds after the 3-month blanking period, occurring during the 12-month follow-up period. Here, we list the patients and relevant data regarding the AF recurrences.

Patient study ID	AF recurrence after the index procedure	Type of recurrent AF	Method of AF detection
03	11 month	paroxysmal	preexisting DDD pacemaker memory
20	5 month	persistent	12 lead ECG due to symptoms
35	12 month	persistent	Holter ECG
37	11 month	persistent	12 lead ECG due to symptoms
38	6 month	paroxysmal	12 lead ECG due to symptoms
40	8 month	paroxysmal	12 lead ECG due to symptoms
